# *Filifactor alocis* and Tumor Necrosis Factor-Alpha Stimulate Synthesis of Visfatin by Human Macrophages

**DOI:** 10.3390/ijms22031235

**Published:** 2021-01-27

**Authors:** Andressa Vilas Boas Nogueira, Marjan Nokhbehsaim, Anna Damanaki, Sigrun Eick, Christian Kirschneck, Agnes Schröder, Jonathan Jantsch, James Deschner

**Affiliations:** 1 Department of Periodontology and Operative Dentistry, University Medical Center, University of Mainz, 55131 Mainz, Germany; a.nogueira@uni-mainz.de (A.V.B.N.); adamanak@uni-mainz.de (A.D.); 2 Section of Experimental Dento-Maxillo-Facial Medicine, Center of Dento-Maxillo-Facial Medicine, University of Bonn, 53111 Bonn, Germany; m.saim@uni-bonn.de; 3 Laboratory of Oral Microbiology, Department of Periodontology, School of Dental Medicine, University of Bern, CH-3010 Bern, Switzerland; sigrun.eick@zmk.unibe.ch; 4 Department of Orthodontics, University of Regensburg, 93053 Regensburg, Germany; christian.kirschneck@ukr.de (C.K.); agnes.schroeder@ukr.de (A.S.); 5 Institute of Clinical Microbiology and Hygiene, University Hospital Regensburg, 93053 Regensburg, Germany; jonathan.jantsch@ukr.de

**Keywords:** periodontitis, *Filifactor alocis*, tumor necrosis factor, visfatin, macrophage, COX2, MMP1

## Abstract

There is little known about the effect of the periodontopathogen *Filifactor alocis* on macrophages as key cells of the innate immune defense in the periodontium. Therefore, the aim of the present study was to investigate the effect of *F. alocis* and additionally of the pro-inflammatory cytokine tumor necrosis factor-alpha (TNFα) on visfatin and other pro-inflammatory and proteolytic molecules associated with periodontitis in human macrophages. The presence of macrophage markers CD14, CD86, CD68, and CD163 was examined in gingival biopsies from healthy individuals and periodontitis patients. Human macrophages were incubated with *F. alocis* and TNFα for up to 2 d. The effects of both stimulants on macrophages were determined by real-time PCR, ELISA, immunocytochemistry, and immunofluorescence. *F. alocis* was able to significantly stimulate the synthesis of visfatin by human macrophages using TLR2 and MAPK pathways. In addition to visfatin, *F. alocis* was also able to increase the synthesis of cyclooxygenase 2, TNFα, and matrix metalloproteinase 1. Like *F. alocis*, TNFα was also able to stimulate the production of these proinflammatory and proteolytic molecules. Our results highlight the pathogenetic role of *F. alocis* in periodontal diseases and also underline the involvement of visfatin in the aetiopathogenesis of periodontitis.

## 1. Introduction

Periodontitis is a highly prevalent inflammatory disease of the tooth-supporting tissues and is caused by dysbiosis of the subgingival microbiome [1,2]. It manifests clinically by deep periodontal pockets, increased tooth mobility, and even tooth loss. The destruction of soft and hard periodontal tissues induced by periodontal pathogens is mediated by the host immune system [2,3]. Periodontal infection leads to the recruitment of a variety of immunoinflammatory cells, among which macrophages play a key role [4,5]. An important property of macrophages is phagocytosis of pathogens, cell debris, and apoptotic host cells [5,6]. They can also present bacterial antigens to lymphocytes, thereby activating the adaptive immune response, and play an important role in the recruitment of other immunoinflammatory cells [5,6]. Macrophages produce a large number of proinflammatory mediators, such as tumor necrosis factor-alpha (TNFα) and prostaglandin E2 (PGE2) produced by cyclooxygenase-2 (COX2), as well as proteases, such as matrix metalloproteinase 1 (MMP1) [5,6]. These molecules are critical components of the inflammatory cascade and mediate soft tissue degradation and alveolar bone resorption in periodontitis [5,6,7]. Furthermore, periodontitis is a risk factor for the development of osteonecrosis of the jaws in patients taking antiresorptive drugs [8].

In recent years, numerous studies have been published showing that periodontitis is associated with a variety of diseases of the entire organism. These diseases include, for example, cardiovascular diseases and type 2 diabetes [9,10,11]. Since obesity is an important risk factor for both diseases, the association between obesity and periodontitis has also been studied [12,13]. It has been shown that there is indeed an association between obesity and periodontitis, and it is believed that obesity may contribute to the development and progression of periodontitis [14]. One mechanism by which obesity may negatively affect the periodontium is adipokines, which are cytokines produced by adipocytes and other cells, such as macrophages, in adipose tissue [15]. In obesity, the number of macrophages in adipose tissue is increased, which leads to increased release of adipokines, such as visfatin, contributing to adipose tissue inflammation [16]. Visfatin, also known as pre-B-cell colony-enhancing factor and nicotinamide phosphoribosyltransferase, plays a role in metabolic regulation as well as immunoinflammatory and wound healing processes [17]. Elevated serum levels of visfatin have been found in obesity, diabetes mellitus, cardiovascular disease, and metabolic syndrome [18,19,20].

Remarkably, increased levels of visfatin have also been detected in serum, saliva, gingival crevicular fluid (GCF), and gingival biopsies from patients with periodontitis compared to periodontally healthy individuals, suggesting that visfatin is also produced in the periodontium [21,22,23]. Moreover, visfatin seems to play a crucial role in the pathogenesis of periodontitis, as suggested by a number of studies [15,22,24]. For example, our group demonstrated that periodontal ligament cells and gingival fibroblasts can produce visfatin, and that its synthesis is increased by *Fusobacterium nucleatum* and *Porphyromonas gingivalis*, two well-studied “classical” periodontopathogens [22,25]. In addition, we found elevated visfatin levels in gingival biopsies from sites with periodontitis [22]. More significantly, however, we provided evidence that visfatin induces the production of chemotactic and proteolytic molecules by periodontal ligament (PDL) cells and thus may contribute to periodontal inflammation and destruction [24]. Interestingly, visfatin also appears to impair the regenerative capacity of PDL cells and therefore periodontal healing, as shown in another study by our group [26]. Overall, these results suggest that visfatin appears to play an important role in the aetiopathogenesis of periodontitis. Further studies are needed to better understand the effects and regulation of visfatin, which in turn could lead to new avenues in periodontal diagnosis and therapy.

The oral microbiome includes more than 700 bacterial species, and several have been associated with periodontal disease [27]. One of the periodontal pathogenic bacteria that has not been extensively studied compared to the classical periodontopathogens is *Filifactor alocis*. *F. alocis* is a Gram-positive anaerobic bacterium and has been detected in saliva and supra- and subgingival biofilm of periodontal patients [28,29,30]. It interacts with other bacteria, particularly *P. gingivalis*, and thus may increase the invasive capacity of this key pathogen [31]. However, little is known about the effect of *F. alocis* on macrophages as key cells of the innate immune defense in the periodontium. Therefore, the aim of this in vitro study was to investigate the effects of *F. alocis* on the synthesis of visfatin and other periodontium-associated molecules, i.e., COX2, TNFα, and MMP1, by human macrophages. It was hypothesized that *F. alocis* stimulates human macrophages to produce visfatin and other proinflammatory mediators associated with periodontitis, thus acting like “classical” periodontal pathogens and inflammatory mediators. A better understanding of the pathogenic role of visfatin and *F. alocis* in the aetiopathogenesis of periodontitis could lead to new diagnostic and preventive strategies.

## 2. Results

### 2.1. Macrophage Infiltration in Human Gingival Tissues from Sites of Periodontitis

**Figure 1:**
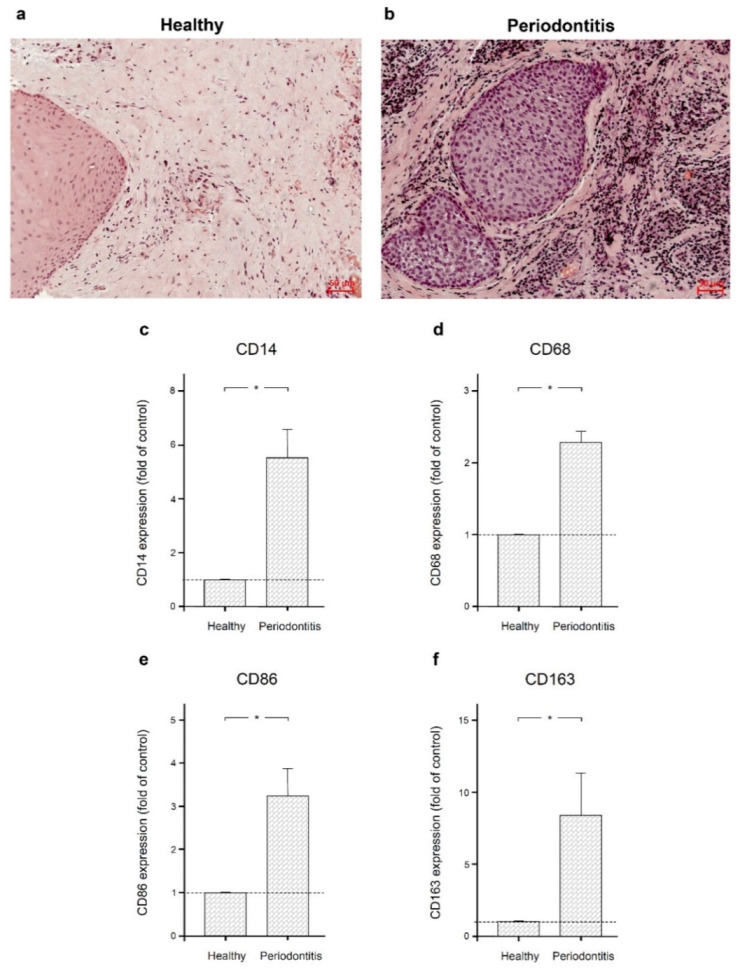
Gingival biopsies from periodontally healthy individuals and periodontitis patients. Gingival biopsies from a periodontally healthy individual (**a**) and a periodontitis patient (**b**). Representative histologic sections are shown. Expression of macrophage markers CD14 (**c**), CD68 (**d**), CD 86 (**e**), and CD163 (**f**) in gingival biopsies from six periodontally healthy subjects and six periodontitis patients. Bars show mean ± SEM. * significant (*p* < 0.05) difference between groups.

As shown in Figure 1a,b, gingival biopsies from periodontitis patients showed a distinct immunoinflammatory infiltrate compared to gingival samples from periodontally healthy individuals. The infiltrate included macrophages, neutrophils, and lymphocytes. To confirm the presence of macrophages, we also examined the expression of the macrophage markers CD14, CD68, CD86, and CD163 in gingival biopsies from periodontally healthy individuals and periodontitis patients. Significantly (*p* < 0.05) increased expression of all these markers was found in inflamed gingiva compared to healthy gingiva, as shown by real-time PCR (Figure 1c–f).

### 2.2. Stimulatory Effects of F. alocis on Human Macrophages

**Figure 2:**
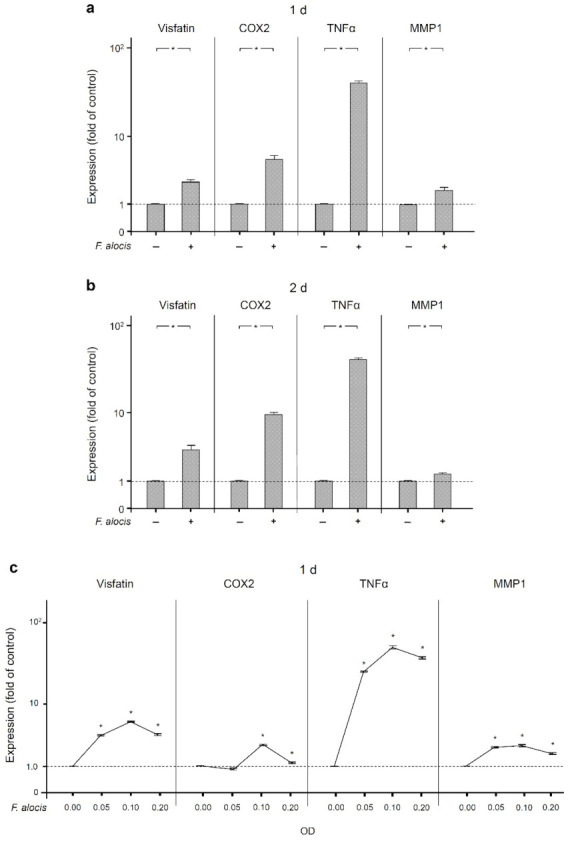
Expression of visfatin, COX2, TNFα, and MMP-1 in the presence and absence of *F. alocis* (OD_660_: 0.1) in human macrophages at 1 d (**a**) and 2 d (**b**), analyzed by real-time PCR. Mean ± SEM, n = 9/group. * significant (*p* < 0.05) difference between groups. Stimulation of visfatin, COX2, TNFα, and MMP-1 expressions by various concentrations of *F. alocis* (OD_660_: 0.05, 0.1, 0.2) in human macrophages at 1 d, as analyzed by real-time PCR (**c**). Mean ± SEM, *n* = 3/group. * significantly (*p* < 0.05) different from control. Unstimulated cells served as control.

Next, we investigated whether *F. alocis* is able to induce the production of molecules associated with periodontal disease. As shown in Figure 2a,b, the expression of visfatin was significantly (*p* < 0.05) upregulated by *F. alocis* in human macrophages after 1 d and 2 d. Similarly, COX2, TNFα, and MMP1 expressions in response to *F. alocis* were significantly (*p* < 0.05) increased at these time points (Figure 2a,b). The *F. alocis*-induced upregulation of visfatin, COX2, TNFα, and MMP1 was dose-dependent (Figure 2c).

**Figure 3:**
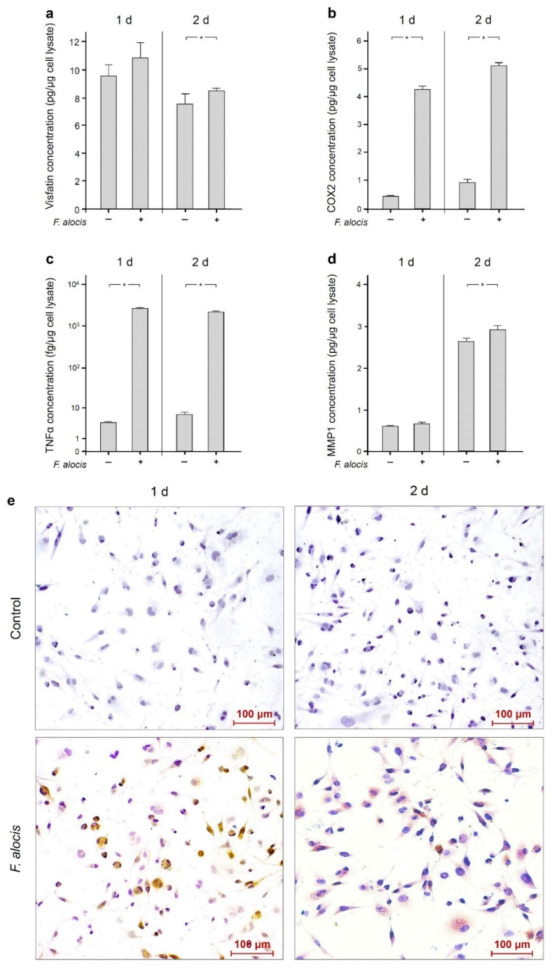
Visfatin (**a**), COX2 (**b**), TNFα (**c**), and MMP-1 (**d**) protein levels in the presence and absence of *F. alocis* (OD_660_: 0.1) after 1 d and 2 d, analyzed by ELISA. Mean ± SEM, n = 18/group. * significant (*p* < 0.05) difference between groups. Visfatin protein in the presence and absence of *F. alocis* (OD_660_: 0.1) in human macrophages at 1 d and 2 d as visualized by immunocytochemistry (**e**). Shown are representative images from one of three experiments.

Moreover, the upregulation of visfatin by *F. alocis* at the transcriptional level was also observed at the protein level at 1 d and 2 d, as analyzed by ELISA (Table 1, Figure 3a). Similarly, increased protein levels of COX2, TNFα and MMP1 were found in *F. alocis*-stimulated macrophages compared to control (Table 1, Figure 3b–d). The differences were significant (*p* < 0.05) for COX2 and TNFα at 1 d and 2 d and for visfatin and MMP1 at 2 d (Table 1, Figure 3a–d).

Moreover, the stimulatory effect of *F. alocis* on visfatin protein was also demonstrated by immunocytochemistry at 1 d and 2 d, as depicted in Figure 3e.

### 2.3. Involvement of Toll-Like Receptor 2 and Intracellular Pathways in the Actions of F. alocis

**Figure 4:**
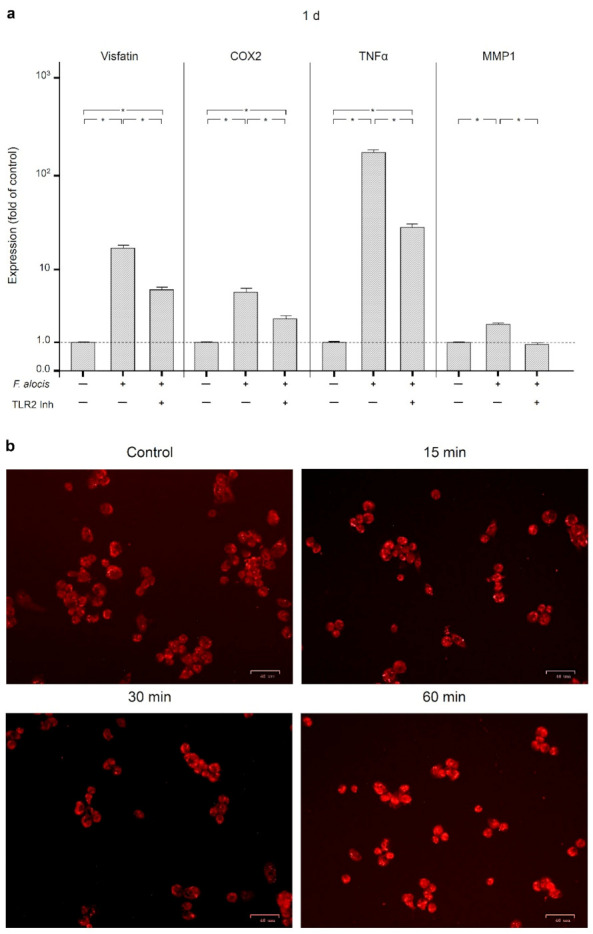
Expression of visfatin, COX2, TNFα, and MMP-1 in response to *F. alocis* (OD_660_: 0.1) in the presence and absence of anti-TLR2 blocking antibody in human macrophages at 1 d, as analyzed by real-time PCR (**a**). Mean ± SEM, n = 3/group. * significant (*p* < 0.05) difference between groups. NF-κB (p65) nuclear translocation in human macrophages stimulated or not with *F. alocis* (OD_660_: 0.1) over 60 min, as examined by immunofluorescence microscopy (**b**). Shown are representative images from one of three experiments.

We next investigated how the effects of *F. alocis* on visfatin, COX2, TNFα, and MMP1 are triggered and subsequently mediated intracellularly. Therefore, we first analyzed whether the effects of *F. alocis* are dependent on toll-like receptor 2 (TLR2). After pre-incubation of human macrophages with an anti-TLR2 blocking antibody, the stimulatory effect of *F. alocis* on the visfatin expression was significantly (*p* < 0.05) reduced at 1 d, as shown in Figure 4a. Likewise, COX2, TNFα, and MMP1 expressions in human macrophages exposed to *F. alocis* were significantly (*p* < 0.05) decreased by blocking TLR2 (Figure 4a).

Since it is known that activation of TLR2 triggers nuclear factor kappa B (NF-κB) signaling, we next examined p65 nuclear translocation in human macrophages stimulated with *F. alocis*. As expected, *F. alocis* caused p65 nuclear translocation, which was most pronounced at 60 min, as analyzed by immunofluorescence microscopy (Figure 4b).

**Figure 5:**
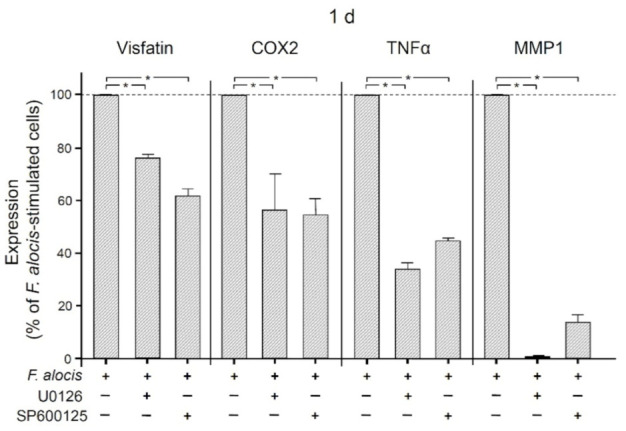
Expression of visfatin, COX2, TNFα, and MMP-1 in response to *F. alocis* (OD_660_: 0.1) in the presence and absence of an inhibitor against MEK1/2 (U0126; 10 𝜇M) or JNK (SP600125; 10 𝜇M) in human macrophages after 1 d, analyzed by real-time PCR. Mean ± SEM, n = 3/group. * significant (*p* < 0.05) difference between groups.

The involvement of MAP/ERK kinases 1/2 (MEK1/2) and c-Jun N-terminal kinases (JNK) signaling was then examined by pre-incubating human macrophages with specific inhibitors against these signaling molecules. As shown in Figure 5, inhibition of MEK1/2 or JNK resulted in significant (*p* < 0.05) down-regulation of *F. alocis*-induced visfatin expression at 1 d. Similarly, significantly (*p* < 0.05) reduced COX2, TNFα, and MMP1 expression levels were detected at 1 d when *F. alocis*-stimulated macrophages were pre-incubated with these inhibitors (Figure 5).

### 2.4. Stimulatory Effects of TNFα on Human Macrophages

**Figure 6:**
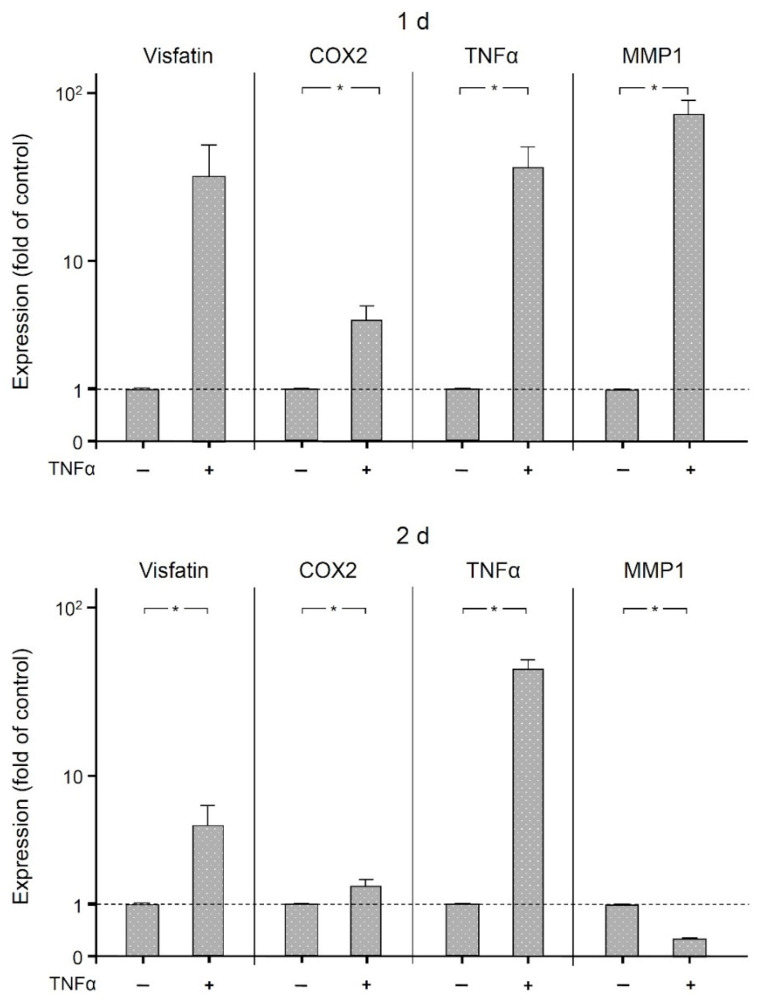
Expression of visfatin, COX2, TNFα, and MMP-1 in the presence and absence of TNFα (1 ng/mL) in human macrophages at (1 d) and (2 d), analyzed by real-time PCR. Mean ± SEM. * significant (*p* < 0.05) difference between groups.

**Figure 7:**
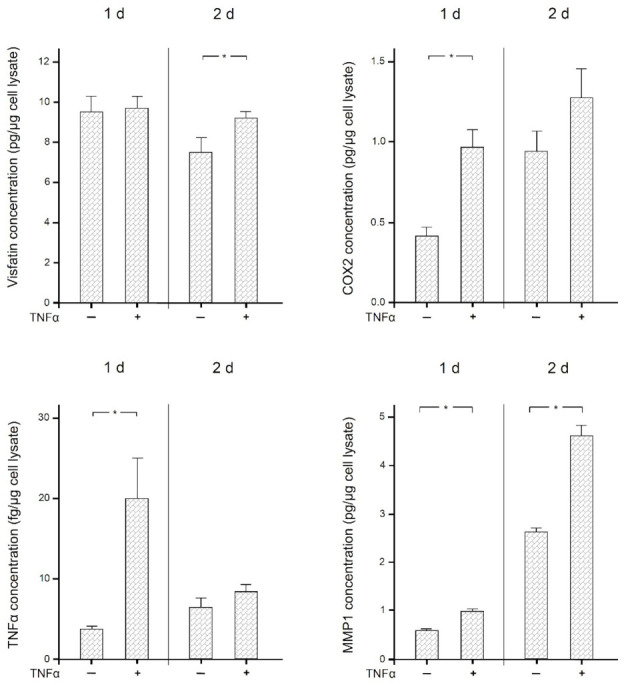
Visfatin, COX2, TNFα, and MMP-1 protein levels in the presence and absence of TNFα (1 ng/mL) at 1 d and 2 d, analyzed by ELISA. Mean ± SEM, *n* = 18/group. * significant (*p* < 0.05) difference between groups.

Finally, we examined the effects of the proinflammatory mediator TNFα on the expression of visfatin, COX2, MMP1, and itself in human macrophages. As shown in Figure 6, TNFα significantly (*p* < 0.05) increased the expression of COX2 and itself at 1 d and 2 d, of MMP1 at 1 d, and of visfatin at 2 d. The stimulatory effect of TNFα on all these molecules was also observed at the protein level as analyzed by ELISA (Figure 7). Significantly (*p* < 0.05) increased protein concentrations were found for MMP1 at 1 d and 2 d, for COX2 and TNFα at 1 d, and for visfatin at 2 d.

## 3. Discussion

Our study shows for the first time that *F. alocis* is able to stimulate the synthesis of visfatin in human macrophages. To do this, *F. alocis* utilizes the TLR2 and MAPK pathways. In addition to visfatin, *F. alocis* was also able to increase the synthesis of COX2, TNFα, and MMP1. Like *F. alocis*, TNFα was also able to stimulate the production of these proinflammatory and proteolytic molecules. Our results highlight the distinct pathogenetic role of *F. alocis* in the development and progression of periodontitis. Furthermore, our results support the important role of visfatin in periodontal infection and inflammation.

*F. alocis* is a Gram-positive, asaccharolytic, anaerobic microorganism strongly associated with periodontal disease, as our group and other investigators have recently shown [28,29,30,31,32,33]. This bacterium can be detected in increased numbers both in dental biofilm and in the saliva of patients with periodontitis [28,29,30]. *F. alocis* is characterized by high resistance to oxidative stress, which promotes its survival and persistence in periodontitis [31]. *F. alocis* cooperates with *P. gingivalis* in biofilm formation and invasion of epithelial cells [31,34]. *F. alocis* exhibits a variety of virulence factors and can stimulate immunoinflammatory and structural cells to release proinflammatory mediators and proteases, as shown in the present investigation and our previous studies [30,31,32,33]. These molecules play key roles in soft tissue degradation and bone resorption in periodontitis [35,36]. Therefore, the results of the present study are consistent with our previous data suggesting a negative effect of *F. alocis* on periodontal health.

The fact that *F. alocis* can upregulate visfatin gene expression and protein synthesis in human macrophages is an exciting finding because the pathogenetic role of visfatin in periodontitis is not yet fully understood and deciphered. Elevated levels of visfatin in serum, saliva, GCF, and gingival biopsies have been observed in periodontitis patients [21,22,23]. We have shown that periodontal cells can produce visfatin and that its production is increased by “classical” pathogens such as *F. nucleatum* and *P. gingivalis* [22,25]. We also demonstrated that visfatin can upregulate the production of chemotactic and proteolytic molecules in PDL cells, which could lead to increased inflammation and more severe tissue destruction in periodontitis [24]. Further studies are needed to better understand the potential effects and regulation of visfatin in periodontal diseases.

In our study, macrophages exposed to *F. alocis* synthesized increased levels of COX2. COX2 is an inducible enzyme produced by cells, for example, during inflammation [37]. It can be induced by bacteria and inflammatory mediators, as in the present study. This enzyme converts arachidonic acid to PGE2, which can then lead to tissue destruction by modulating connective tissue metabolism [38]. COX2 is associated with periodontal disease [35,39]. Elevated levels of COX2 and PGE2 have been found in saliva, gingival biopsies, and gingival crevicular fluid from patients with periodontitis [40,41,42,43,44], indicating the critical role of this enzyme in the aetiopathogenesis of periodontitis. Further evidence for the key role of COX2 in periodontitis comes from studies that have shown the adjunctive benefit of COX2 inhibitors in the therapy of periodontitis [45,46,47].

In the present in vitro study, *F. alocis* caused an upregulation of MMP1 in macrophages. MMPs are proteinases involved in tissue formation, remodeling, and degradation; i.e., MMPs play a crucial role under physiological and pathophysiological conditions [48]. In periodontitis, MMP1, which is a collagenase, plays a key role in periodontal soft tissue destruction and alveolar bone loss [36]. It can cleave types I and III collagen fibers, which are the most abundant components of the periodontal tissue matrix [36,48]. Elevated levels of MMP-1 have been found in GCF and gingival tissue samples from periodontitis sites compared to periodontally healthy sites [49,50,51]. In addition, MMP1 levels have been found to be decreased after periodontal treatment [52,53], highlighting the pathophysiological role of this collagenase in periodontal disease conditions.

*F. alocis* also stimulated macrophages to produce increased levels of TNFα, a proinflammatory cytokine strongly associated with periodontal inflammation and destruction. This inflammatory mediator has been shown to promote the expression of adhesion molecules and chemokines, the production of other inflammatory mediators and proteases, and the formation and activity of osteoclasts, thereby contributing to periodontal inflammation, soft tissue degradation, bone loss, and impaired periodontal repair [54,55]. Therefore, it has been suggested that TNFα in GCF could serve as a potential biomarker for the diagnosis of periodontal diseases [56].

In the present study, we also stimulated macrophages with TNFα. Like *F. alocis*, TNFα increased the expression and synthesis of visfatin, COX2, MMP1, and itself. These results confirm the established role of TNFα in periodontal inflammation and destruction. On the other hand, however, they underscore the proinflammatory role of *F. alocis*, which exerted the same effects as the inflammatory mediator TNFα in our study on macrophages.

Our experiments showed that the effects of *F. alocis* on visfatin, COX2, MMP1, and TNFα expression were TLR2-dependent. Moreover, pre-incubation of macrophages with specific inhibitors showed that the proinflammatory and proteolytic actions of *F. alocis* also involved the mitogen-activated protein kinase (MAPK) pathway. The present results are consistent with our previous studies in fibroblastic and monocytic cells, in which we also demonstrated that the actions of *F. alocis* are dependent on TLR2 and the MAPK pathway [32,33]. Interestingly, interaction of *F. alocis* with TLR2 was reported to induce granule exocytosis along with MAPK activation in neutrophils [57]. Moreover, *F. alocis*-derived extracellular vesicles activated TLR2 downstream signaling of the MAPK and NF-κB pathways in mouse bone-derived mesenchymal stromal cells [58]. The findings of these studies are consistent with our results in that *F. alocis* appears to utilize TLR2 and the MAPK pathway for its effects. Whether lipoteichoic acid and/or peptidoglycan, which are cell wall components of *F. alocis*, interacted with TLR2 to trigger the actions of *F. alocis* should be investigated in future studies. Further studies are needed to better understand the intracellular signaling pathways that mediate the effects of *F. alocis* on macrophages.

In the present study, macrophages were incubated with *F. alocis*. However, periodontitis is a complex disease characterized by a variety of different bacteria [27]. Future studies should also focus on the combined effects of *F. alocis* with other periodontopathogens on macrophage cells. In addition, the effects of *F. alocis* alone or in combination with other biofilm bacteria on co-cultures of macrophages with periodontal structural cells, such as gingival fibroblasts, would be a very exciting area of research.

In summary, our study provides original evidence that *F. alocis* is able to stimulate the synthesis of visfatin in human macrophages via TLR2 and MAPK pathways. *F. alocis* also increased the production of COX2, TNFα, and MMP1. Therefore, our results highlight the pathogenetic role of *F. alocis* in the initiation and progression of periodontitis. Furthermore, our findings also underline the important role of visfatin in periodontal infection and inflammation.

## 4. Materials and Methods

### 4.1. Culture and Treatment of Cells

A human acute monocytic leukemia cell line (THP-1) (CLS Cell Lines Service, Eppelheim, Germany) was used. THP-1 suspension cells were pretreated with phorbol 12-myristate 13-acetate (PMA, 100 nM; Calbiochem, San Diego, CA, USA) for 1 d and differentiated into adherent macrophages. In pre-experiments, different PMA concentrations (50 nM, 100 nM, 200 nM, and 1000 nM) were tested, and the best concentration was 100 nM of PMA, which had also been used by other investigators [59,60]. Macrophages (1 × 10^5^ cells) were grown in RPMI 1640 culture medium (Invitrogen, Karlsruhe, Germany) supplemented with 10% fetal bovine serum (FBS, Invitrogen), 100 units penicillin (Invitrogen), and 100 μg/mL streptomycin (Invitrogen) at 37 °C in a humidified atmosphere of 5% CO_2_. Cell culture medium was renewed every two days. One day prior to each experiment, the FBS concentration was reduced to 1%. Macrophages were stimulated with the inactivated periodontopathogen *F. alocis* ATCC 3589 (optical density, OD: 0.05, 0.1, and 0.2) for up to 2 d. The strain was pre-cultivated on Schaedler agar plates (Oxoid, Basingstoke, UK) in an anaerobic atmosphere for 2 d. Subsequently, *F. alocis* was suspended in phosphate-buffered saline (PBS, Invitrogen) (OD_660_ = 1, equivalent to 1.2 × 10^9^ bacterial cells/mL). Afterwards, the bacteria suspension was twice ultrasonicated (160 W for 15 min), occasioning complete bacterial killing. To unravel signaling mechanisms by which *F. alocis* might modulate the expression of inflammatory mediators, cells were pre-incubated with specific inhibitors of intracellular signaling pathways (10 μM; U0126 for MEK1/MEK2; P600125 for JNK; Calbiochem), 1 h prior to the *F. alocis* stimulation. Cells were also pre-incubated with a blocking anti-human TLR2 monoclonal antibody (10 µg/mL; eBioscience, San Diego, CA, USA) 45 min prior to *F. alocis* stimulation. Moreover, cells were stimulated with human recombinant TNFα (Biomol, Hamburg, Germany). TNFα was applied at a concentration of 1 ng/mL, which had also been used by other investigators [61]. Untreated cells served as control.

### 4.2. Human Gingival Biopsies

Human gingival biopsies were collected in the Department of Oral Surgery at the University of Bonn as follows: Healthy gingiva was obtained from healthy gingival sites of six periodontitis-free patients during wisdom tooth surgery, whereas inflamed gingiva was derived from sites with periodontitis of six patients in whom teeth had to be extracted for periodontal reasons. Periodontally healthy sites were characterized by the following parameters: gingival index = 0 (no clinical inflammation), periodontal pocket depths ≤ 3 mm, and no clinical attachment and radiographic bone loss. Periodontitis sites were characterized by gingival index ≥ 1 (clinical inflammation), periodontal pocket depths ≥ 5 mm, and clinical attachment and radiographic bone loss [1]. Approval of the Ethics Committee of the University of Bonn and written informed consent by the patients or their parents were obtained (#043/11). The exclusion criteria were the presence of smoking and/or systemic diseases. Six gingival biopsies from each group were stored immediately in RNA stabilization reagent (RNAlater) (Qiagen, Hilden, Germany) and kept in the −80 °C freezer until use. Additional gingival tissues from both groups were fixed in 4% paraformaldehyde (Sigma-Aldrich, Munich, Germany) for 2 d, hydrated, and dehydrated in an ascending ethanol series (AppliChem, Darmstadt, Germany). Subsequently, they were embedded in paraffin (McCormick Scientific, Richmond, IL, USA), sliced in tissue sections of 2.5 µm thickness, mounted on glass slides (Engelbrecht, Edermünde, Germany), and dried at 37 °C overnight. Afterwards, all sections were stained with hematoxylin and eosin (H&E, Merck Eurolab, Darmstadt, Germany), dehydrated, and mounted with DePeX (SERVA Electrophoresis GmbH, Heidelberg, Germany). Finally, the presence or absence of gingival inflammation was analyzed in the stained tissue sections using Axioskop 2 microscope (Carl Zeiss, Jena, Germany) with an AxioCam MRc camera and the AxioVision 4.7 software (Carl Zeiss).

### 4.3. Real-Time PCR

RNeasy Mini Kit (Qiagen) was used to extract total RNA according to the manufacturer’s protocol. Afterwards, RNA concentration was determined with NanoDrop ND-2000 (Thermo Fisher Scientific, Wilmington, DE, USA) spectrophotometer. Five hundred ng of total RNA was reversely transcribed using the iScript™ Select cDNA Synthesis Kit (Bio-Rad Laboratories, Munich, Germany) at 42 °C for 90 min and 85 °C for 5 min, following the manufacturer’s instructions. PCR amplification was performed using an iCycler iQ™ Real-Time PCR Detection System (Bio-Rad), SsoAdvanced™ Universal SYBR^®^ Green Supermix (Bio-Rad), and specific primers (QuantiTect Primer Assay, Qiagen). Expressions of visfatin, COX2, TNFα, MMP1, CD14, CD68, CD86, CD163, and glyceraldehyde-3-phosphate dehydrogenase (GAPDH) was analyzed by quantitative RT-PCR. Reaction mixture contained 1 µL of cDNA, 12.5 µL of SsoAdvanced™ Universal SYBR^®^ Green Supermix (Bio-Rad), 2.5 µL of primers, and 9 µL RNase free water. The heating protocol used was initially at 95 °C for 5 min, followed by 40 cycles of a denaturation step at 95 °C for 10 s, and a combined annealing/extension phase at 60 °C for 30 s. The comparative threshold cycle method was used for the gene expression analysis.

### 4.4. ELISA

Protein levels of COX2 were analyzed in macrophage cell lysates using a commercially available detection kit (DYC4198-2; human/mouse total COX2 ELISA with DuoSet, R&D Systems Europe, Abingdon, United Kingdom). Protein levels of visfatin (human NAMPT ELISA Kit, SK00121-01, Hölzel Diagnostika, Cologne, Germany), TNFα (human TNF-α Quantikine ELISA Kit, DTA00C, R&D Systems, Wiesbaden-Nordenstadt, Germany), and MMP1 (human MMP-1 ELISA Kit, RayBiotech, Norcross, GA, USA) were measured in macrophage cell culture supernatants. The protocols were followed according to the manufacturer’s instructions for each specific kit. The concentration of visfatin, COX2, TNFα, and MMP-1 was measured by spectrophotometry using a microplate reader (PowerWave X, BioTek Instruments, Winooski, VT, USA). Pierce BCA Protein Assay Kit (23227, Thermo Fisher Scientific) was used to measure the total protein concentrations and normalize the data.

### 4.5. Immunocytochemistry

Cells were grown on 13 mm diameter plastic coverslips (Thermo Fisher Scientific) in a 24-well plate. Cells were stimulated or not with *F. alocis* (OD: 0.1) for 1 d and 2 d and stained for the presence of visfatin. Cell monolayers were fixed in 4% paraformaldehyde (Sigma-Aldrich) at pH 7.4 and room temperature for 10 min and then permeabilized in 0.1% Triton X-100 (Sigma-Aldrich) for 5 min. Subsequently, cell monolayers were blocked with serum block (Dako, Hamburg, Germany) for 20 min followed by incubation with rabbit polyclonal primary antibody to visfatin (ab45890, 1:200; Abcam, Berlin, Germany) in a humid chamber at 4 °C overnight. Later, cells were incubated with goat anti-rabbit IgG HRP secondary antibody (Dako) for 45 min. Then, cells were stained with DAB chromogen (Dako) in the dark for 10 min. Cells were washed twice with PBS after each incubation stage. Afterwards, Mayer’s Hematoxylin (Merck Eurolab) was used for counterstaining, and the coverslips were mounted in DePeX mounting medium (SERVA Electrophoresis). Finally, photomicrographs were taken with Axioskop 2 (Carl Zeiss) equipped with a 20 × objective, and images were acquired with an AxioCam MRc camera (Carl Zeiss) and AxioVision 4.7 software (Carl Zeiss).

### 4.6. Immunofluorescence

Cells cultured on plastic coverslips (Thermo Fisher Scientific) were stimulated or not with *F. alocis* (OD: 0.1) for 60 min. Afterwards, the cells were fixed and permeabilized, as mentioned above, and blocked with nonfat dry milk (Bio-Rad) at room temperature for 1 h. Next, the cells were incubated with primary rabbit anti-NF-𝜅B p65 antibody (E498, 1:100, Cell Signaling Technology, Danvers, MA, USA) for 90 min, rinsed with PBS and then incubated with secondary CY3-conjugated goat anti-rabbit IgG antibody (1:1000, Abcam) for 45 min. Cells were washed twice with PBS after each incubation step. Cells were examined under a 20 × objective using the ZOE™ Fluorescent Cell Imager (Bio-Rad). Images were captured using an integrated 5MP digital CMOS camera.

### 4.7. Statistical Analysis

Statistical analysis was performed using IBM SPSS Statistics software (Version 22, IBM SPSS, Chicago, IL, USA). Normal distribution of the data was tested with the Shapiro–Wilk test. Mean values and standard errors of the mean (SEM) were calculated. Statistically significant (*p* < 0.05) differences between the groups were identified by *t*-test, Mann–Whitney U test, or ANOVA followed by the post-hoc Tukey’s or Dunnett’s multiple comparison tests. All experiments were performed in triplicate and repeated at least twice.

## 5. Conclusions

Our study provides original evidence that *F. alocis* is able to stimulate the production of visfatin and other proinflammatory and proteolytic molecules by human macrophages, using TLR2 and MAPK pathways. *F. alocis* and visfatin may play a critical pathogenetic role in the development and progression of periodontitis.

## Figures and Tables

**Table 1:** Protein levels in supernatants (visfatin, TNFα, and MMP1) and cell lysates (COX2) of human macrophages treated with *F. alocis*.

	1 Day		2 Days	
	Control	*F. alocis*	Control	*F. alocis*
Visfatin (pg/µg)	9.52 ± 0.77	10.81 ± 1.10	7.50 ± 0.73	8.45 ± 0.17 ^1^
COX2 (pg/µg)	0.42 ± 0.05	4.22 ± 0.11 ^1^	0.94 ± 0.13	5.05 ± 0.10 ^1^
TNFα (fg/µg)	3.87 ± 0.27	2619.51 ± 58.26 ^1^	6.52 ± 1.11	2151.72 ± 83.37 ^1^
MMP1 (pg/µg)	0.61 ± 0.02	0.67 ± 0.05	2.64 ± 0.07	2.92 ± 0.10 ^1^

^1^ significant (*p* < 0.05) difference compared to control. Mean ± SEM, *n* = 18/group.

## Data Availability

Data sharing is not applicable to this article.

## Abbreviations

ATCC	American Type Culture Collection
cDNA	complementary desoxyribonucleic acid
COX2	cyclooxygenase-2
ELISA	enzyme-linked immunosorbent assay
FBS	fetal bovine serum
GAPDH	glyceraldehyde-3-phosphate dehydrogenase
GCF	gingival crevicular fluid
HRP	horseradish peroxidase
JNK	c-Jun N-terminal kinases
MAPK	mitogen-activated protein kinase
MEK1/2	MAP/ERK kinases 1/2
MMP1	matrix metalloproteinase 1
NF-κB	nuclear factor kappa B
OD	optical density
PCR	polymerase chain reaction
PDL	periodontal ligament
PGE2	prostaglandin E2
PMA	phorbol 12-myristate 13-acetate
RNA	ribonucleic acid
TLR2	toll-like receptor 2
TNFα	tumor necrosis factor-alpha
